# Influence of environmental enrichment on rodents’ brains: neurochemical and neuroanatomical aspects

**DOI:** 10.3389/fvets.2026.1767287

**Published:** 2026-05-07

**Authors:** Adriana Domínguez-Oliva, Ismael Hernández-Avalos, Antonio Bueno-Nava, Cuauhtémoc Chávez, Antonio Verduzco-Mendoza, Dina Villanueva-García, Alberto Avila-Luna, Adriana Olmos-Hernández, Julio Martínez-Burnes, Arturo Gálvez-Rosas, Daniel Mota-Rojas

**Affiliations:** 1 Programa de Doctorado en Ciencias Biológicas y de la Salud, Universidad Autónoma Metropolitana, Mexico City, Mexico; 2 Neurophysiology, Behavior and Animal Welfare Assessment, DPAA, Universidad Autónoma Metropolitana, Mexico City, Mexico; 3 Facultad de Estudios Superiores Cuautitlán (FESC), Universidad Nacional Autónoma de México (UNAM), Cuautitlán, Mexico; 4 Division of Neurosciences, Instituto Nacional de Rehabilitación-Luis Guillermo Ibarra Ibarra (INR-LGII), Mexico City, Mexico; 5 Departamento de Ciencias Ambientales, CBS Universidad Autónoma Metropolitana-Lerma, Lerma, Mexico; 6 Department Bioterio and Experimental Surgery, Instituto Nacional de Rehabilitación-Luis Guillermo Ibarra Ibarra (INR-LGII), Mexico City, Mexico; 7 Division of Neonatology, Hospital Infantil de México Federico Gómez, Mexico City, Mexico; 8 Facultad de Medicina Veterinaria y Zootecnia, Instituto de Ecología Aplicada, Universidad Autónoma de Tamaulipas, Victoria City, Mexico

**Keywords:** dopamine, mice, neurogenesis, norepinephrine, rat, serotonin

## Abstract

Housing laboratory rodents in complex and enriched environments increases their exposure to physical, sensorial, social, and cognitive stimuli. Exposure to enriched conditions has been associated with animals having an optimized response to cope with challenges. This is related to improvements in cognitive functions, including memory and learning, processes that are modulated by the monoaminergic system. Dopamine, serotonin, and norepinephrine participate in several functions, including alertness, motivation, arousal, motor control, emotional regulation, and neuronal plasticity. Environmental conditions influence their concentration in different brain regions, and research has shown that environmental enrichment improves the monoaminergic system regulation. Furthermore, neurochemical adaptations are also accompanied by changes in brain structure, as well as promoting neurogenesis and enhancing synaptic activity. This review presents an overview of the studies addressing the effect that environmental enrichment has on the neurochemistry and neuroanatomy of laboratory rats and mice. It will focus on the monoaminergic system to summarize the changes that enriched conditions elicit in dopamine, serotonin, and norepinephrine concentrations in different brain regions. Additionally, it will discuss some neuroanatomical aspects and differences in the electrical brain activity in laboratory rodents housed in enriched conditions.

## 1 Introduction

Environmental conditions influence the physiological, behavioral, and emotional state of laboratory rodents (1–4). The experience that animals obtain from their environment has significant effects on neurochemistry and brain plasticity (2, 5–7). Restricted or impoverished living environments (e.g., small cages with bedding only) are often related to stress responses by limiting social interactions, physical exercise, and mental stimulation (8). In contrast, complex environments have been shown to induce synaptic plasticity and cognitive improvements (7, 9). Environmental enrichment (EE) has been defined as a method “to enhance animal well-being by providing animals with sensory and motor stimulation, through structures and resources that facilitate the expression of species-typical behaviors and promote psychological well-being through physical exercise, manipulative activities, and cognitive challenges according to species-specific characteristics” (10, 11). It is differentiated from conventional housing because this type of housing generally meets the minimum standard for laboratory rodents, providing cages roughly adequate for the animal size, containing food, water, and absorbent bedding (10, 12). For rodents, physical, sensorial, social, and cognitive EE is provided through group-housing in larger cages with manipulable toys, running wheels, tunnels, or objects to gnaw (7, 13–15). These enrichments increase the complexity of the environment and provide greater opportunities for rodents to interact with novel stimuli (7). By improving the spatial complexity, several studies have reported a significant effect on cognitive functions, including memory and learning (5, 6).

These cognitive processes are highly dependent on neurochemical levels, particularly the monoaminergic system (16, 17). This system is a network of neurons in the nervous system that uses neurotransmitters, such as dopamine (DA), serotonin (5-HT), and norepinephrine (NE), to modulate functions including alertness, motivation, arousal, cognition, motor control, emotional regulation, and neuronal plasticity (18–22). Thus, the ability of rodents to appropriately respond to their environment is a dynamic process where monoamine levels play a crucial role (23). For example, studies have shown that the extracellular concentration of DA in the prefrontal cortex (PFC) of mildly stressed rats has a significantly lower increase in enriched rats, which might reflect a downregulation of the stress response modulated by the mesocortical dopaminergic system (24, 25), Moreover, a diminished DA metabolism and the production of its main metabolite, 3,4-dihydroxyphenylacetic acid (DOPAC), have been associated with the greater ability of enriched animals to cope with novel external stimuli (26–28). However, monoamine levels could reflect altered baseline arousal or motivational state that may not always be beneficial.

The complexity of the environment can also alter 5-HT and NE levels. In enriched animals, an increase in 5-HT and NE hippocampal concentrations has been reported (20–22). In particular, the hippocampus is a brain area susceptible to environmental aspects (29, 30). Therefore, hippocampal monoamine levels have been studied –and are emphasized in the present review– due to the serotonergic and noradrenergic innervation of the hippocampus, as well as its participation in spatial navigation, learning, and memory (23). Moreover, EE enhances hippocampal plasticity, a process modulated by monoamines (21). Regardless of the brain region, EE has been shown to modulate functional monoamine neurotransmission at basal levels or during exposure to stressors, which is a trait necessary for animals to cope with challenges (21, 22, 24, 25, 31).

Housing laboratory rodents in enriched conditions increases their opportunities for social and exploratory activity, and this has shown a significant effect on brain structure (13, 32, 33). The neuroanatomical changes associated with EE include an increased brain mass, cortical thickness, neurogenesis, and synaptic profiles, among others (20, 34). EE is known to influence neuroplasticity by neurogenesis in the dentate gyrus of the hippocampus (13, 15, 29, 35, 36).

Changes in neurotransmitter levels and brain modifications differ highly according to the evaluated structure, and the function of each structure must be considered to understand the benefits of EE in rodent brains. This review presents an overview of the studies addressing the effect that EE has on the neurochemistry and neuroanatomy of laboratory rats and mice. It will focus on the monoaminergic system to summarize the changes that EE elicits on DA, 5-HT, and NE levels in different brain regions. Additionally, it will discuss some neuroanatomical aspects and differences in the electrical brain activity in laboratory rodents housed in enriched conditions.

## 2 Search strategy

A literature search was conducted using Web of Science, Scopus, and PubMed from August 2025 to September 2025. The keyword search items included the following combination of words: “laboratory rodents,” “rats,” “mice,” “environmental enrichment,” “monoamine,” “dopamine,” “serotonin,” “norepinephrine,” “noradrenaline,” “brain structure,” “neurogenesis,” and “synaptic changes.” The initial search yielded over 2000 papers. Further refinement of the search was performed by two reviewers through the inclusion/exclusion criteria. Included studies were original research papers where rats/mice/gerbils received EE and described the type of enrichment. Selected studies incorporated EE in healthy animals or in those exposed to an acute stressor or stress paradigm, without inducing any disease. Since the literature reporting monoamine changes in healthy animals is limited, a publishing date exclusion was not established to summarize the findings. Only journal papers in English were selected. Review papers were cited when used as a physiological basis for the monoaminergic system or when discussing the neurotransmitter changes. Exclusion criteria considered studies adopting EE in rodents serving as an animal model for any disease (e.g., brain injury). Studies with genetically modified rodents where a specific gene or receptors were inactivated or deleted were excluded. Non-full text. After filtering the studies according to the inclusion/exclusion criteria, 57 papers were included in the present review.

## 3 Monoamine changes related to enriched environments

Monoamines (DA, 5-HT, and NE) are neurotransmitters that regulate a wide range of functions, including mood, cognition, sleep, and attention (16). They participate in basic physiological processes (e.g., cardiovascular and respiratory system) and during the processing of complex cognitive and emotional activities. They influence the so-called incentive motivation that is altered due to housing conditions (37), as the ability to cope with environmental challenges is a dynamic process where the monoamine system is required to rapidly respond to external stimuli (23).

**Figure 1:**
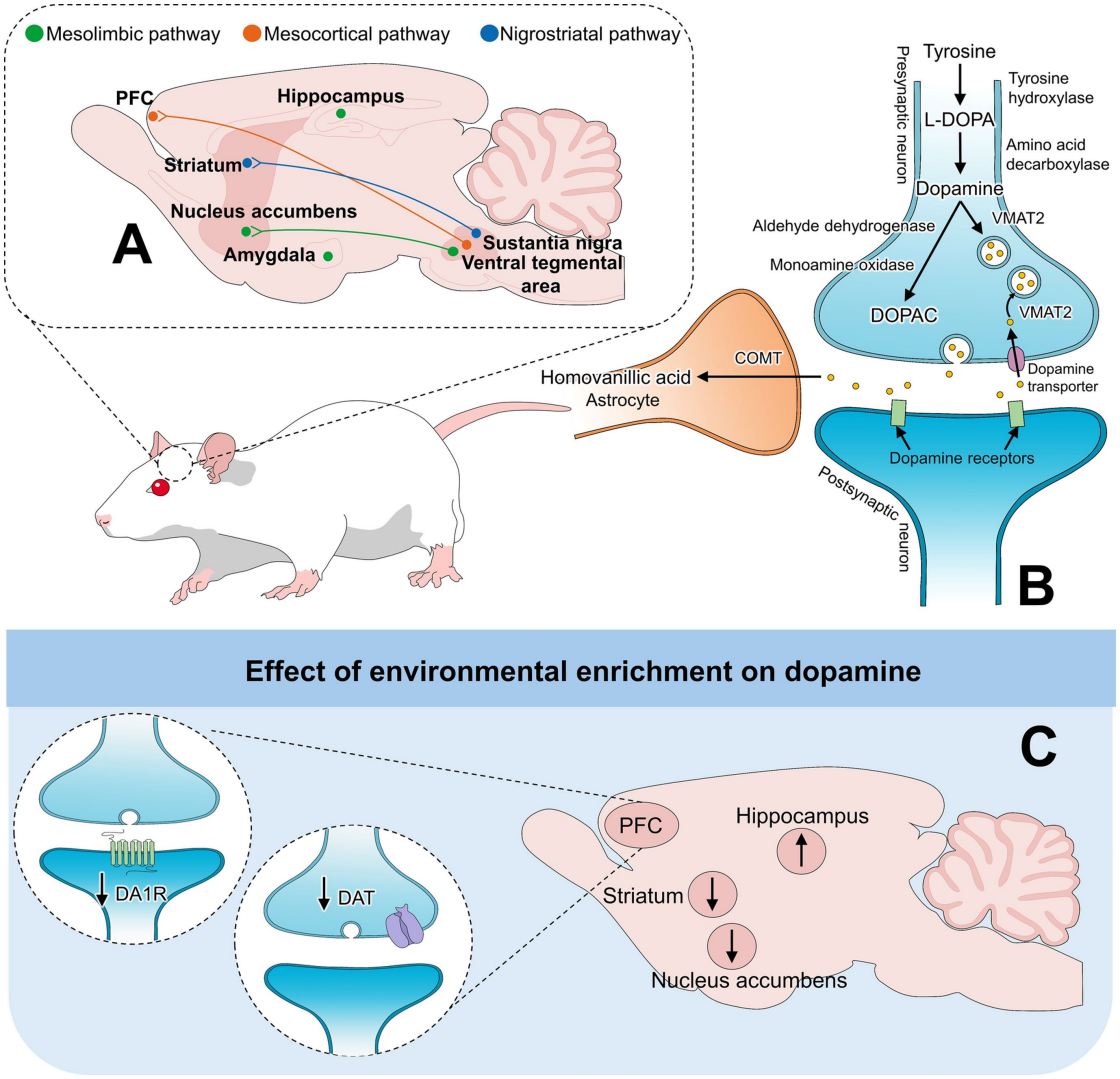
Dopamine pathways and synthesis. **(A)** Main dopamine pathways: the mesolimbic, mesocortical, and nigrostriatal. The first two pathways project dopaminergic fibers from the VTA to the NAc or the PFC, respectively. In contrast, nigrostriatal pathways project from the SN to the striatum. **(B)** Dopamine synthesis and metabolism into the main DA metabolite DOPAC. **(C)** Effects of EE on dopamine pathways. ↑: reported an increase in at least one EE study in that region; ↓: reported a decrease in at least one EE study in that region. These symbols may aggregate heterogeneous and sometimes conflicting findings. COMT: catechol-o-methyltransferase; DA1R: dopamine receptor 1; DAT: dopamine transporter; DOPAC: 3,4-dihydroxy-phenylacetic acid; PFC: prefrontal cortex; VMAT2: vesicular monoaminergic transporter-2.

The most prevalent catecholamine in the Central Nervous System (CNS) is DA (17). It modulates neuronal signaling involved in cognitive function, motivation, and reward behaviors (18, 19). EE has been shown to modify the concentration of DA, particularly those involving dopamine projections to the nucleus accumbens, prefrontal cortex (PFC), and hippocampus due to its role in cognition and motor behavior (9). Dopaminergic pathways and synthesis have been schematized in Figure 1 (9, 38–41).

Segovia et al. (24) reported significantly higher *in vivo* extracellular concentrations of DA in the nucleus accumbens of male Wistar rats (3 months) reared in enriched conditions for 6 months (by 80%). EE consisted of large metacrilate cages (housing 10–12 animals) with running wheels, plastic tunnels, elevated platforms, and toys, while standard animals were individually housed. Moreover, enriched animals showed a decreased novelty-induced locomotor activity. This reflects an enhanced mesolimbic DA system in enriched conditions, which is associated with a faster habituation to novel environments. Following the same enrichment program, another study from the same authors reported that EE significantly influenced the release of DA in the PFC of male Wistar rats (3 months old) (25). After animals reached 6 months, the authors used handling to evoke mild acute stress and assessed the effect of rearing conditions and stress on DA concentrations. It was found that stress significantly increased the dialysate concentration of DA in the PFC of both enriched (average of 1.00 nM) and control animals (average of 1.25 nM). However, enrichment ameliorated this increase, which the authors associate with a lower stress response and downregulation of the mesocortical DA system.

Del Arco et al. (27) reported similar findings when evaluating DA and acetylcholine (ACh) extracellular concentrations through microdialysis in the medial PFC (mPFC) of male Wistar rats (3 months of age), housed in either enriched or control conditions for 12 months. EE was provided in large methacrylate cages (10–12 animals per cage) with running wheels, plastic tunnels, elevated platforms, and toys. To assess the responses to stress and monoamine changes, 40 min of acute mild stress (handling) was applied to animals. Basal dialysate concentrations of DA and ACh were not significantly different between control (0.85 ± 0.2 and 44.3 ± 6.4 nM, respectively) and enriched conditions (0.53 ± 0.1 and 38.1 ± 8.1, respectively). During handling stress, although DA concentrations significantly increased in both housing conditions, there were no significant differences. In contrast, stress significantly increased the extracellular levels of ACh in the mPFC of control rats (from 44.6 ± 5.7 to 61.7 ± 8.3 nM), but not in enriched rats. DA can stimulate ACh release by acting on cholinergic neurons in the nucleus accumbens. Thus, a lower DA release during stress is related to lower ACh release in the PFC. Moreover, these results, together with lower motor activity in the open field test, suggest that enriched rats had lower reactivity to stress in the cholinergic but not dopaminergic system in the mPFC due to their higher ability to cope with stressors and new environments. Better coping during an acute stress challenge was also recorded in male Wistar rats (3 months old) housed under enriched conditions (large cages, group housing, running wheels, tunnels, elevated platform, and objects) (28). When animals reached 6 months of age, microdialysis concentrations of DA and corticosterone in the PFC were evaluated during acute restraint stress (20 min). Enriched animals had significantly lower increases of DA (approximately 110%) and corticosterone (150%), in contrast to isolated rats (approximately 170 and 250%, respectively). These results suggest that EE mitigates the reactivity to stress by downregulating the hypothalamic–pituitary axis and the mesocortical dopaminergic system. However, a limitation of the findings is that neither study considered other stress indicators, such as fecal corticosterone or physiological parameters (e.g., heart rate).

When measuring tissue concentration, Zhu et al. (26) assessed the concentrations of DA and DOPAC (main DA metabolite) in the mPFC, striatum, and nucleus accumbens of enriched and isolated male Sprague–Dawley rats (PND 21) using a high-performance liquid chromatography system. EE animals were reared in larger metal cages (8–12 animals per cage) with non-chewable plastic objects until 53 days of age. While no significant differences were observed in any brain region for DA, a tendency for lower DA in the mPFC was observed in EE animals (approximately 0.12 *vs*. 0.14 ng/mg tissue). In contrast, DOPAC content in the mPFC was significantly lower in enriched animals (0.075 *vs*. >0.1000 ng/mg tissue). The decrease in DOPAC in the mPFC suggests a decrease in DA metabolism, an aspect that has been associated with the ability of enriched animals to effectively respond to several stimuli (27, 28). Bowling et al. (40) reported a significant reduction of tissue DA in the nucleus accumbens (approximately 2 μg/g tissue) and the striatum (2.5 μg/g tissue) of 21-day-old male Sprague Dawley rats enriched with wire mesh cages with toys. The authors concluded that it might be related to greater dendritic ramifications and higher concentration of non-neural elements in enriched animals when compared to standard or isolated individuals (2). In this regard, Winterfeld et al. (42) reported that enriched housing highly influences DA innervation of the mPFC of male gerbils (PND 90). Animals were group-housed (3–6 animals) in semi-natural, large wooden cages containing tunnels, tubes, branches, and solid hiding places for 60 days. When comparing the maturation of the right prefrontal DA innervation of standard animals (individually housed), enriched gerbils had 56% more fiber density. Additionally, isolated animals had severe working memory impairments.

Not only monoamine concentration but also the expression of DA receptors is influenced by EE. For example, the density of DA 1 (D1) receptors in the PFC of 3-month-old enriched male Wistar rats was evaluated using immunocytochemistry (9). EE was provided during 90 days in large methacrylate cages with running wheels, plastic tunnels, and toys. A significant reduction in D1 receptors in the PFC of EE animals was observed (approximately 400 arbitrary units) when compared to standard animals (700 arbitrary units). The reduction in D1 receptors is associated with a reduction in motor activity, as enriched animals are known to have more efficient exploratory behavior and faster habituation.

Similarly, studies have reported an effect of EE on the expression of DA transporters (DAT), proteins that regulate the amount of DA in the brain by clearing it from the synaptic cleft and returning it to neurons (43). An example is Zhu et al. (44), who reported a decrease in DAT cell surface expression in the mPFC of 21-day-old male Sprague–Dawley rats housed in enriched conditions. Enriched animals were group-housed (8–12 rats per cage) in a large metal cage with non-chewable plastic objects until 53–55 days of age, whereas standard animals were individually housed. No differences in DAT expression were reported in the nucleus accumbens and striatum. However, the expression of DAT significantly decreased in the mPFC by 39%. Kim et al. (41) mention that the alterations in the dopaminergic system due to EE are related to internalization of striatal DAT. This was studied in 6-week-old male CD-1 mice housed in large cages (10 mice/cage) with tunnels, shelters, toys, and running wheels for 2 months. Using positron emission tomography imaging, the authors found that striatal DA transporter binding values decreased by 18%, showing a lower uptake than control animals (housed in standard cages). Moreover, internalization of DA transporters was observed, resulting in 30% lower expression of membrane DAT by phosphorylation.

**Figure 2:**
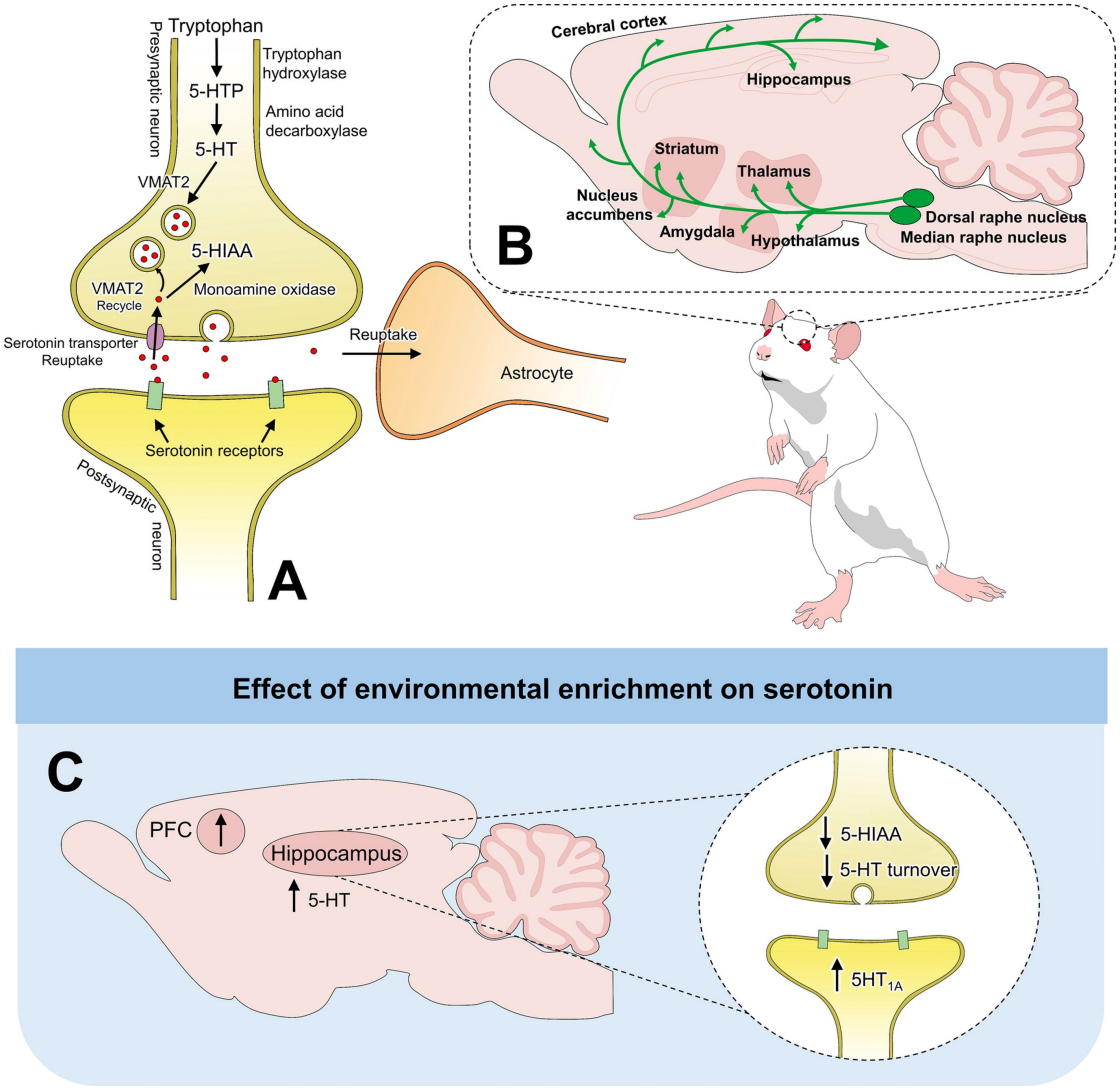
Serotonin pathways and synthesis. **(A)** 5-HT synthesis and metabolism into the main 5-HT metabolite, 5HIAA **(B)** Main serotonergic projections innervating the Hyp, Amy, thalamus, and striatum, among other brain regions. **(C)** Effects of EE on serotonergic pathways. ↑: reported an increase in at least one EE study in that region; ↓: reported a decrease in at least one EE study in that region. These symbols may aggregate heterogeneous and sometimes conflicting findings. 5-HT: serotonin; 5HTP: 5-hydroxytryptophan; 5-HIAA: 5-Hydroxyindoleacetic acid; 5HT1A: 5-HT 1A receptor; PFC: prefrontal cortex; VMAT2: vesicular monoaminergic transporter-2.

Similar to the effect that EE has on DA tissue concentrations, other studies have focused on 5-HT and NE to determine how the environment modifies the neurochemical response of animals. In the first instance, 5-HT is a neurotransmitter that regulates mood, memory, and stress responses, among other functions. Serotoninergic pathways and synthesis have been schematized in Figure 2 (21, 31, 45–47). It also has a fundamental role in neural development and plasticity of the CNS by modulating cognitive functions (48). Moreover, 5-HT is implicated in neurogenesis, learning, and memory (29), processes that are influenced by EE (20, 48).

**Figure 3:**
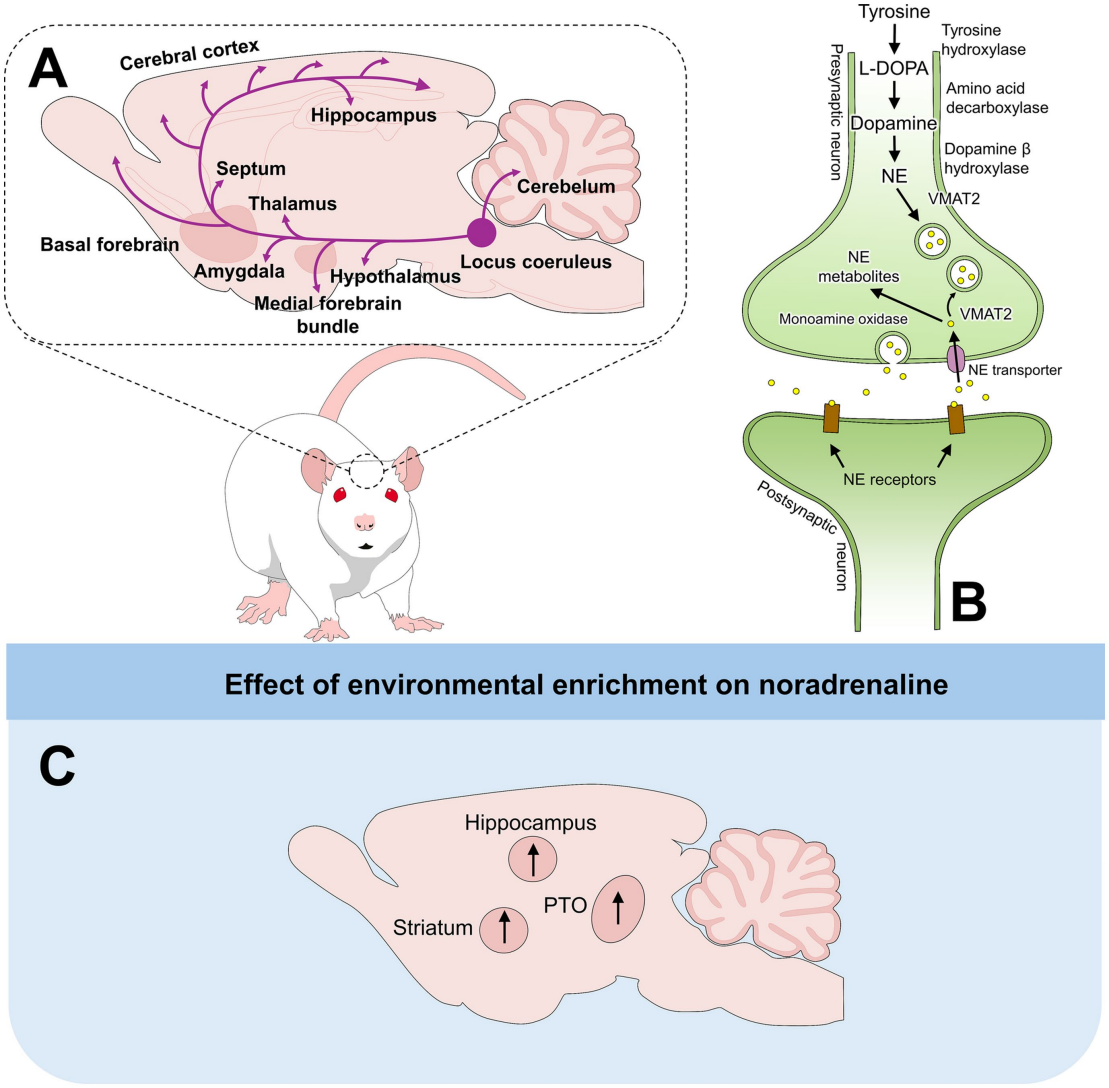
Noradrenergic innervation and synthesis. **(A)** Main noradrenergic projections innervating the Hyp, medial forebrain bundle, Amy, thalamus, and the entire cerebral cortex, among other brain regions. **(B)** NE synthesis and metabolism. **(C)** Effects of EE on noradrenergic pathways. ↑: reported an increase in at least one EE study in that region. These symbols may aggregate heterogeneous and sometimes conflicting findings. NE: norepinephrine; PTO: parieto-temporo-occipital cortex; VMAT2: vesicular monoaminergic transporter-2.

On the other hand, NE or noradrenaline facilitates several neural processes, such as synaptic plasticity, and enhances learning and memory by activating beta-adrenergic receptors (49). The noradrenergic system (schematized in Figure 3, (21, 23, 49, 50)) modulates cognitive function, arousal, and emotion and plays a crucial role in regulating attention (51). Moreover, noradrenergic fibers actively participate in the “fight or flight” response elicited during stress.

Some studies have evaluated the integral response of the monoaminergic systems, as shown by Guan et al. (23), who determined the concentrations of DA, 5-HT, and NE in the hippocampus of rat offspring (PND 21) exposed to either an enriched or standard environment after being subjected to a chronic unpredictable mild stress. The enriched conditions included group housing (four rats per cage) in larger cages with ramps, running wheels, shelters, and among other objects, for a period of 30 days. The standard or control group was group-housed in conventional cages. In contrast to standard animals, rats in the enriched group recorded a significant increase in DA (up to 400 μg/mL), 5-HT levels (up to 450 μg/mL), and NE (approximately 250 μg/mL). Moreover, these increases were accompanied by a significant decrease in corticosterone (200 ng/mL), which is related to a diminished stress response. Increased 5-HT concentrations (together with neurotrophins) facilitate the formation of synapses in the CNS (21). Synaptic formation is necessary for mammals to habituate to novel environments, and EE is known to promote rapid habituation to novel environments and reduce stress response, in which the monoamine system participates (21).

In another study assessing the effect of EE (larger cages with non-chewable plastic objects and PVC tubes) on tissue levels of right hippocampal 5-HT and NE in male Sprague–Dawley rats (PND 28), Brenes et al. (31) reported significant differences when compared to animals housed in standard (SC) and isolation (SI) conditions. The EE group had significantly higher tissue levels of 5-HT (0.04 ng/mg of wet tissue) and NE (approximately 0.06 ng/mg of wet tissue) than SC and SI groups (both less than 0.02 ng/mg of wet tissue). The author also reported significant differences regarding 5-HT turnover, where EE had the lowest value, while SI was significantly higher. 5-HT turnover, a parameter which refers to the rate at which 5-HT is synthesized, released, and broken down, is an important measurement since it maintains normal brain function and regulates mood. In this aspect, studies have shown that repeated exposure to a stressor (e.g., elevated open platform) significantly increases hippocampal 5-HT turnover so animals are able to habituate to the stressor (52). Regarding 5-HT turnover, contradictory findings were reported by Galani et al. (48), who evaluated adult female Long-Evans rats (12 weeks old) exposed to enriched conditions and standard housing. Enrichment lasted 30 days and consisted of large wire-mesh cages with 12 rats/cage with various objects (not specified). Monoamine concentrations were determined in the frontoparietal cortex and the hippocampus. EE reduced the 5-HT levels in the ventral hippocampus by 23%, increased 5-HT turnover in the entire hippocampus by 36%, and increased NE levels in the dorsal hippocampus by 68%. No significant differences were found in the frontoparietal cortex or for DA levels according to the housing conditions. The authors also found that when evaluating defecation in the elevated plus-maze, droppings had a negative correlation with 5-HT (r = −0.36) and NE (r = −0.41), which suggested the relationship between EE, the neurochemical response, and emotional states (anxiolytic effects).

Hippocampal concentrations of 5-HT and NE have also been shown to correlate with antidepressant behaviors, as reported by Brenes et al. (21). When comparing swimming behavior (evaluated through the forced swimming test, FST) of male Sprague–Dawley rats (PND 28) exposed to EE, positive and strong correlations were found with hippocampal 5-HT concentrations (r = 0.84). Moreover, EE rats had negative and strong correlations between 5-HT and NE with immobility (r = −0.80). In the FST, an increase in active behaviors (swimming) and a decrease in passive ones (immobility) reflect an antidepressant-like effect (53), a response that is related to the increase in 5-HT and NE in the hippocampus (21).

Neurochemical changes (5-HT and corticosterone) associated with EE were also determined in the hippocampus, frontal cortex, and hypothalamus of C57Bl6/N mice (12–15 weeks old) (54). Enriched animals received red, transparent plastic igloos, plastic tunnels, and nesting material (tissue). In contrast, standard conditions include bedding in conventional cages. While the levels of 5-HT were not significantly different between groups in the hippocampus, frontal cortex, and hypothalamus, levels of 5-hydroxyindoleacetic acid (5-HIAA), the primary metabolite of 5-HT, were significantly lower in the hypothalamus of enriched male mice (401.2 ± 13.6 vs. 452.1 ± 18.8 pg./mg wet weight). This finding is relevant because it suggests a downregulation of hypothalamic serotonergic function due to EE. Moreover, as Rasmuson et al. (47) mention, EE can influence serotonergic receptors (5HT_1A_) in the dorsal hippocampus by increasing their expression by 44%–62%. In this study, male Sprague–Dawley rats (PND 52) were exposed to EE or single housing for 30 days. Enriched rats were group-housed (4 animals per cage) in wire mesh cages containing objects and toys for 30 days. To measure 5-HT1A receptor expression, the authors performed autoradiographic localization of hippocampal receptors in coronal sections of the dorsal hippocampus. Enriched rats had significantly higher 5-HT_1A_ receptor mRNA expression in the dorsal hippocampus (up to 62%). These findings might be related to the role of hippocampal 5-HT_1A_ role for learning and memory (55).

Concentrations of 5-HT and NE were also evaluated in the PFC and ventral striatum (VS) of male Sprague–Dawley rats (PND 30) reared in larger cages with non-chewable plastic objects and PVC tubes (21). After 84 days of EE, when compared to SC (standard cages with bedding) and SI animals, enriched rats had significantly higher 5-HT levels in the PFC (0.02 ± 0.01 ng/mg) and NE in the VS (0.11 ± 0.02). Additionally, although no data is shown in the study, the authors reported that the PFC of enriched rats was significantly heavier than standard and isolated rats, which might be related to more 5-HT fibers innervating the PFC in enriched rats. In male mice of the strain ICR (PND 28), Naka et al. (49) evaluated the effect that EE has on monoaminergic neurons by comparing standard and enriched conditions. The enriched condition consisted of group-housed mice with access to larger plexiglass cages for 3 h daily. The cages contained plastic tubes, a small house with a ladder, running wheels, and toys, while the standard mice were housed in groups (3–4 mice per cage) in standard cages. After 40 days of EE, the authors evaluated the concentrations of DA, 5-HT, and NE on the left side of the frontal cortex, parieto-temporo-occipital cortex, hippocampus, cerebellum, and pons through HPLC. Although no significant differences were found for DA and 5-HT in any brain region, the authors reported a whole-brain increase of 10.7% in NE concentration. Particularly, in the parieto-temporo-occipital cortex, the concentration of NE increased by 27.3% in comparison with the standard group. Likewise, increases by 37.5 and 17.0% in the cerebellum and pons, respectively, were reported for NE. The higher concentrations of NE are related to the physiological function of the neurotransmitter by regulating synaptic plasticity and enhancing learning and memory, aspects that are implicit in enriched environments. Supplementary Table S1 summarizes the characteristics of EE provided to rodents in each discussed study and the main neurochemical outcomes.

### 3.1 Integration of behavior and neuroendocrine outcomes

Changes in the monoaminergic system are frequently associated with behavioral modifications in rodents. An example is the antidepressant-like effect attributed to EE. This has been observed in male Sprague Dawley rats reared in enriched conditions for 84 days, in whom higher levels of 5-HT and NE in the PFC and VS were correlated with active behaviors such as swimming, climbing, and diving (21). Similarly, higher hippocampal concentrations of 5-HT in enriched Sprague Dawley males were strongly correlated with swimming behavior (31). In the FST, active behaviors can be elicited by antidepressant drugs that selectively inhibit NE and 5-HT uptake (56). Thus, it could be suggested that EE can have an antidepressive-like effect and even induce an anxiolytic-like effect, where EE has been shown to restore hippocampal levels of 5-HT by 0.7-fold (57).

Enriched environments are also associated with accelerated locomotor habituation. This was observed in male Sprague Dawley and Wistar rats housed under EE and evaluated using the open-field test (27, 31). Enriched rats traveled the shortest distance at a slow speed, and they also displayed less thigmotactic scanning (31), which suggests that rats were less fearful and willing to explore new environments (27, 58). Novelty-seeking behavior was also observed in female Long-Evans rats reared in enriched large wire-mesh cages, who had a high percentage of open arms entries (up to 50%) in the elevated plus-maze (48). This behavior suggests anxiolytic effects or a rapid habituation to stress (14). Additionally, 5-HT and 5-HIAA levels in the ventral hippocampus, and NE in the dorsal hippocampus were negatively correlated with the number of droppings (r = −0.36 and −0.41, respectively) (48). It is known that acute psychological distress induces defecation in rodents via the hypothalamus-raphe magnus-spinal axis (59). Thus, a decrease in the number of droppings and increased monoamine concentrations suggest that EE might have anxiolytic effects due to the role that 5-HT and NE have on anxiety-related behavior.

In enriched rats, it has also been observed that animals spend significantly more time grooming (70% of the time licking the body) and are more engaged in this behavior than animals housed in standard conditions and social isolation (31). This might be an index of habituation in response to novel environments. Although no correlation between monoamines, grooming, and EE has been reported, dopaminergic pathways modulate self-grooming in rodents (60). For instance, in male and female mice, the mesolimbic pathway increases grooming presentation and duration, while inputs projecting to the dorsomedial striatum (nigrostriatal) have the contrary effect (61). Furthermore, studies have suggested that EE can increase emotional reactivity, animal curiosity, and pleasure, and that these responses are probably linked to modifications in hippocampal DA, 5-HT, and NE (23). Research on healthy male and female individuals at different developmental stages is still required to comprehensively evaluate how EE influences monoaminergic pathways and the effect on innate, social, and affiliative behaviors.

## 4 Neuroanatomical changes associated with enriched environments

The brain is a dynamic organ whose structure and function are highly influenced by the environment (13, 32, 62). Apart from the neurochemical changes associated with cognitive improvements, the provision of EE to rodents has been shown to modify anatomical traits such as the morphology of brain regions, adult neurogenesis, neuron survival, and neuroplasticity, among other beneficial effects (33, 63–65). In particular, changes in hippocampal neurons are critical since the neuroplasticity of the hippocampus is dependent on the environment (29, 35).

Regarding the morphology of the brain, Diamond et al. (66) reported changes in the cortical depth when comparing enriched *vs*. impoverished male rats of the S1 strain. The animals receiving EE were housed in large cages (10–12 rats/cage) with access to ladders, tunnels, and swings, among other objects, for 30 days. The results indicated that enriched rats had more extensive cortical depth, particularly in the dorsal cortical segments. Moreover, the caudal region of the cerebral cortex, including the occipital cortex, recorded the greatest difference according to the housing conditions. In other studies by the same authors (2, 67), structural changes influenced by the environmental conditions have been documented in rats. Enriched rats (strain and age are not specified) were housed in groups in a large cage with wooden toys, a wooden maze, and access to the Hebb-Williams maze. Through the ocular micrometer method, it was found that the cortical depth of the enriched brains was 6.4% greater than that of standard (impoverished environment) rats. Likewise, the mean depth of the visual cortex of enriched animals was 6.2% significantly larger, while standard animals had significantly fewer neurons in the somatosensory area (7% per microscopic field). Therefore, it was concluded that the environmental experience of animals influences the depth of the cortex and, possibly, cognitive traits.

Similar benefits on the volume of specific anatomical regions have been evaluated by performing *in vivo* magnetic resonance imaging (MRI) on male C57BL/B6 mice (seven-week-old) exposed to EE (68). Enrichment was provided during 3 weeks by adding domes, running wheels, and an adjustable three-level maze that could be rearranged to create new pathways. The findings showed that the volume of regions associated with the sensorimotor function and spatial navigation increased by 10% in enriched animals, including the ventral hippocampus, entorhinal cortex, and subiculum. Significantly larger somatosensory and visual cortices, as well as in the left/right striatum, hypothalamus, and ventral tegmental area (VTA), were found in EE animals. Additionally, by considering the findings of *ex vivo* MRI performed by the same authors (68), it is suggested that novel experiences elicited with EE modulate brain plasticity as early as 24 h after exposure to the enrichment.

The results found by Scholz et al. (68) are relevant as the brain regions where differences were reported are key components related to EE. For example, cognitive processes involving learning, navigation, exploration, and locomotion are modulated by the hippocampus (30, 69). Therefore, changes in its structure reflect the effect that EE has on brain function. In this sense, a significant increase in the number of bromodeoxyuridine (BrdU)-positive cells in the hippocampus of enriched male Sprague Dawley rats (2026.0 ± 118.7 cells/granule cell layer) was reported by Ueda et al. (29), which is related to cell proliferation in the brain elicited by EE. The enlargement of the somatosensory and visual cortex suggests an association between the sensorial experience provided through EE and the structural reorganization of these areas (68). Moreover, the changes in the VTA indicate the activation of the reward system in mice exposed to an enriched environment.

Other researchers have documented the effect that EE has on neurogenesis. In male Fischer aged rats (20–22 months), Speisman et al. (70) housed animals in pairs in large wooden/wire cages containing empty water maze tanks, and toys such as plastic tubes, balls, and various objects for 10 weeks. In contrast, standard conditions consisted of individual housing. The results showed that, although neurogenesis decreased with age, EE improved cell survival and increased the density of new cells in the dentate gyri (enriched: 1345.13 ± 204.12 cells/mm^3^ *vs*. individually housed: 881.10 ± 156.82 cells/mm^3^). Similar results were found in 9-week-old female Sprague–Dawley rats housed either in isolation or under EE (35). During eight weeks, EE was provided with larger cages with climbing ladders, platforms, paper, nesting material, cardboard nests, popcorn, and apples. The total number of BrdU-positive cells in the granule cell layer was determined. A statistically significant increase in the number of BrdU cells was found in EE rats (136.9 6 10.4 cells/mm3) when compared to isolated animals (72.425 6 15.5 cells/mm3). These result suggests that EE promotes the proliferation of neural cells, which might be related to the rapid ability of enriched animals to acquire and use novel information (35, 70).

Monteiro et al. (71) also mention that EE increases neurogenesis in mice exposed to 7 days of either enrichment or isolation. Enriched male Swiss mice (8 to 12 weeks old) were housed in larger cages containing plastic shelters, cardboard rolls, toys, and ribbons. The authors compared isolated animals in both standard and EE conditions, and group-housed animals (five animals per cage) in ST and EE conditions. A significant increase in the number of newborn neurons in the dentate gyrus of the hippocampus and glomerular layer of the olfactory bulb was recorded in group-housed and individually housed mice. Moreover, group housing and EE significantly increased the number of neurons (approximately 600 BrdU/NeuN+nuclei/mm^2^) in contrast to group housing without EE (around 400 BrdU/NeuN+nuclei/mm^2^). Neurogenesis in the dentate gyrus of the hippocampus was also studied by Segovia et al. (32) in male Wistar rats of 2 (young) and 25 (old) months. The animals were housed under EE in large cages during 8 weeks, receiving running wheels, a rearrangeable set of plastic tunnels, an elevated platform, and toys. Standard rats were kept in individual cages. A significantly higher number of BrdU-positive cells in the dentate gyrus was recorded in young rats exposed to EE when compared with standard animals (1,200 *vs*. 800 cells/mm^2^). In old rats, the number of BrdU-positive cells in EE animals was also higher when compared with the control group; however, the difference was not statistically significant (400 vs. 200 cells/mm^2^). Additionally, the authors evaluated dialysate glutamate concentrations, finding that extracellular glutamate levels in the hippocampus of aged rats were significantly increased. These findings suggest that EE not only promotes neurogenesis but might also act as a compensatory mechanism for neuronal deterioration in aged animals. In fact, other studies have highlighted the benefits of combining EE with physical exercise, owing to its survival-promoting effects on newborn cells (72).

Providing running wheels to promote voluntary exercise in young (3 months) and aged mice (19 months) has also shown benefits on hippocampal neurogenesis, as found by van Praag et al. (73). Mice were individually housed with or without a running wheel. After 45 days, it was found that young and aged mice with access to the running wheel had significantly higher numbers of BrdU-positive cells (2,355 and 656, respectively) than sedentary mice (613 and 117 cells, respectively). Similarly, Kempermann et al. (74) found in 21-day-old mice that EE increased the number of neurons in the dentate gyrus of the hippocampus, and increased the hippocampal granule cell layer by 15%.

As Ohline and Abraham (6) mention, EE increases synaptic excitability, particularly of hippocampal neurons in the dentate gyrus. An increased synaptic transmission was observed in male Sprague–Dawley rats (21 months old) housed in large two-story cages containing toys and various objects for 3 hours a day (from 5:00 p.m. to 8:00 p.m.), 7 days a week, for 3 weeks (75). Standard animals were single-housed. Extracellular recordings in gassed artificial cerebrospinal fluid were obtained from the dendritic layer of the CA1 region. In contrast to samples from standard animals, in enriched rats, the authors successfully induced long-term potentiation (159 ± 16% of baseline) and long-term depression (68 ± 3%, open circles), which might be related to an increased glial population due to EE.

Similar findings were reported by Green and Greenough (76) when male Long-Evans rats (PND 22) were exposed to EE with large wire-mesh cages containing three-dimensional objects for 25–34 days. When compared to animals individually housed, EE rats recorded a significantly higher basal synaptic transmission in the hippocampal dentate gyrus and a significantly larger population spikes at different stimulus intensities. The activity of hippocampal neurons is influenced by the complexity and sensorial stimulation provided through the environment. Thus, the significantly higher synaptic responses recorded in enriched animals might reflect an improved acquisition of information. This is related to what Irvine et al. (77) evaluated in freely moving adult male Sprague–Dawley rats implanted with electrodes in the perforant path and dentate gyrus. EE was provided to singly housed rats reared in larger boxes with toys (e.g., plastic drainpipes, tunnels, ladders, and children’s toys) and novel food (Kellogg’s Coco Pops) for 19 days or overnight. Standard animals were housed singly in conventional cages. EE exposure was for 19 days after establishing basal field excitatory postsynaptic potentials (fEPSPs) and population spikes (PSs). In the study, it was found that exposure to EE increased fEPSPs, but only when animals were kept in low-stress conditions. An increase in PSs was also observed, and although all housing conditions induced it, the significantly greater effect was recorded when providing EE overnight, suggesting that EE increases information processing in the hippocampus.

Supplementary Table S2 summarizes the neuroanatomical and neurotransmission changes observed when animals are provided with EE. These changes, together with the neurochemical response of rodents, suggest that complex environments improve the ability of animals to integrate information. This aspect is closely related to the learning and memory processes required to cope with challenges. Providing stimulating environments to laboratory rodents helps them to efficiently respond to several stimuli, diminishing their reactivity (or stress response) by modifying immediate neurochemical aspects such as the monoaminergic system or by inducing long-term changes, such as brain structural modifications.

## 5 Discussion

### 5.1 “Standard” versus “impoverished” housing conditions

Across studies, in addition to EE protocol inconsistencies, the housing conditions of the control group differ greatly. Some studies refer that animals are group-housed in standard cages with bedding material and *ad libitum* water and food (54). However, some control conditions are individual housing with no additional stimulation, which may themselves represent impoverished or stress-inducing conditions, not genuine “standard” husbandry, particularly from a welfare perspective. For example, the European Union has stated in Annex III of the Directive 2010/63/EU that “Establishments shall have appropriate enrichment techniques in place, to extend the range of activities available to the animals and increase their coping activities, including physical exercise, foraging, manipulative and cognitive activities, as appropriate to the species” (78).

Under this scope, most of the control conditions in the reviewed studies could be considered barren housing conditions that might have implications on the neuroendocrine response of laboratory rodents. The provision of these housing conditions not only impacts animal welfare but also creates challenges when comparing results between studies. Furthermore, it needs to be considered that comparing an enriched environment with a deliberately deprived or social isolation condition might exaggerate the apparent benefit of EE. An alternative would be the comparison of a refined but non-enriched housing condition to better approximate incremental welfare improvements relevant to regulatory guidance.

### 5.2 Heterogeneity and reproducibility of EE protocols to study neurochemical and neuroanatomical changes in rodents

The studies included in the present review suggest that EE can regulate the monoaminergic system and promote neurogenesis in laboratory rodents. While every study shares a similar finding, the challenge with EE protocols is the high heterogeneity among studies. As can be seen in Supplementary Tables S1, S2, enrichment characteristics and protocol duration are inconsistent. This complicates standardizing EE protocols for laboratory rodents. For instance, most EE protocols for rodents employ larger cages with different dimensions, providing an average of 1,039 cm^3^ per animal (79). However, this value differs according to the number of animals housed in the cage.

Group size among EE protocols is highly variable, ranging from two to 15 animals (79). This difference makes it challenging to standardize a single group size across laboratories, but it can also influence hierarchy formation, aggression, and affiliative interactions (80). For example, in male BALB/c mice, higher levels of aggression have been observed in groups of eight animals than in groups of three (81). Similar results were reported for male mice of the CD-1 strain (82). For rats, a suggested group size between two and lower than 12 individuals has been reported (83). This is relevant when evaluating the monoaminergic response, as aggression and socio-environmental stress in rodents are associated with lower brain 5-HT levels (84), decreased noradrenergic activity in the hippocampus and hypothalamus, and increased DA turnover in the PFC (85).

Regarding enrichment items, most studies implement structural enrichment, including tunnels, running wheels, and shelters, strategies recommended to promote the expression of species-specific behaviors or fine motor movements (7, 13–15). However, not all protocols describe in detail the type and number of items used. Some refer to the items as “various objects” or “toys” without further specifying. Similarly, although the items are listed, there is frequently no reference to the number of items per cage, which is a methodological limitation to replicability in other laboratories and comparability of neurochemical and structural outcomes across studies. A recent meta-analysis concluded that adding 5 or more resources (e.g., additional space, burrowing substrates, gnawing materials, complex cages, and foraging opportunities, among others) is recommended to reduce morbidity and improve animal welfare (12).

Another aspect that is inconsistent across studies is the frequency of object rotation. While some protocols rotate enrichment items daily (40, 70), others do so weekly (54). This has implications in cognitive and spatial acquisition, as mentioned by Speisman et al. (70), who reported that daily exposure to EE increased hippocampal neurogenesis in aged rats. Although there is no established or preferred rotation frequency, prolonged exposure to an enrichment object might decrease the novelty effect (86), which could influence the effect on rodents’ monoamine system. Furthermore, even though item rotation needs to be considered to avoid habituation to the EE protocol, changing the habitat too frequently might also cause distress to some animals (10).

Other aspects associated with the rotation frequency of enrichment items are the access schedule and duration of the EE protocol. Some studies provide EE continuously (21, 31), while other protocols give access to enriched areas only for 3 h daily (49). The selection seems to depend on methodological bases that are not addressed in the studies. Moreover, there is no consensus on whether continuous or intermittent EE is better for animal welfare, and some research has elucidated neurological differences between EE schedules. For instance, it has been reported that continuous EE for 4 weeks in male and female aged Long-Evans rats prevents cognitive decline and improves spatial memory (87). Similarly, age-related memory decline was significantly reduced in male mice provided with continuous EE for 10 weeks, in contrast to mice exposed to EE only for 3 h per day (88). In contrast, Rojas-Carvajal et al. (89) found that a restricted EE protocol (enrichment provided randomly from two to 48 h for 30 days) upregulates the expression of genes related to neural plasticity in the dorsal striatum and hippocampus.

The duration of the enrichment protocol can vary from 7 days to several months. A review concluded that 40% of studies have a duration of 4–8 weeks, 28% of 1–4 weeks, and only 2% of studies implement EE for 1 year or longer periods (79). It is unclear whether the duration of the protocol could influence neurochemical or neuroanatomical outcomes. However, both the duration and the schedule are parameters that can affect animal response to human interaction. For example, some studies describe that animals are handled daily to replace enrichment items, while animals in standard groups are not handled until evaluations or tissue extraction are required (26, 40). In other instances, enriched and standard animals receive minimal handling during cage cleaning (24, 27). This might be of particular importance in studies that use stress paradigms, where enriched animals may have been more habituated to human contact or manipulations, potentially confounding stress reactivity outcomes. In such cases, EE protocols must be implemented to prevent or minimize the potential influence that handling habituation could have on experimental findings.

### 5.3 Social behavior and housing structure as variables

Most of the revised studies use social and structural enrichments. However, some of them use structural-only enrichment. Thus, although the findings can be reported as a single enrichment protocol, the results could be more accurately described as the effect of “increased social housing” or “enhanced physical activity” rather than truly multimodal EE. When considering the impact that social interaction or structural complexity might have on animals’ neurochemical and neuroanatomical changes, it might be beneficial to individually evaluate each type of enrichment. This has only been evaluated in some instances. An example is Faherty et al. (33) study in mice divided into standard, exercise (standard cage + running wheel), and enriched groups (running wheels + nesting material + tubes). Results showed that hippocampal changes in the CA1 and dentate gyrus were observed only in the enrichment group. This might be related to environmental complexity, novelty, and exploratory behavior, and not an increase in motor activity, as observed in the exercise group. Evaluating each component of environmental enrichment, rather than a protocol, could help to understand the neuroendocrine mechanisms and neuroanatomical changes that EE generates in rodents.

When addressing social enrichment, most studies include social housing as one element of EE rather than a major independent factor. Thus, the specific contribution of social structure versus structural enrichment to the reported monoaminergic and neuroanatomical changes remains unclear. It is known that group housing for rodents is recommended and is considered a type of EE, as they are social species (3, 90). In this sense, some studies have compared isolation without EE versus isolation with EE (71). It was found that, although isolated animals showed less neurogenesis in the olfactory bulb, regardless of the social status, mice provided with EE increased the number of newborn neurons in the dentate gyrus of the hippocampus. This suggests that, rather than the social conditions, environmental complexity has a positive effect on the animals’ neuroanatomy. Further research on the specific contribution of social interaction and structural enrichment on both the monoaminergic system and neuroanatomical changes could help disentangle these components.

### 5.4 Age and sex-specific effects

The age at onset of EE varies considerably across the reviewed studies. It ranges from immediately after weaning (at 21 PND) to 21 months. It has been reported that 41% of studies that use EE in laboratory rodents implement a protocol during the first 1 to 4 weeks of life, while 33% do so at 4–8 weeks (79). This is related to the neuroplastic events that occur mainly in developing brains during the first month of life (4, 79). Moreover, when assessing neurochemical and neuroanatomical outcomes and EE, age is an important factor. In young rodents, the early establishment of an EE protocol has been shown to increase *in vivo* extracellular concentrations of DA in the nucleus accumbens (24) and increase tissue concentration of DA, 5-HT, and NE in the hippocampus (23), which is associated with habituation to novel environments (9). From a welfare perspective, early-life experiences influence cognitive functions and the way animals respond to stimuli. These experiences, even in the neonatal period, enhance hippocampal-dependent learning and persist into adulthood (91), which is relevant for both welfare and translational research.

In the case of EE and aged rats and mice, research is focused on preventing cognitive impairments, although approximately 2% of studies include animals older than 1 year (79). For instance, ageing physiologically decreases hippocampal neurogenesis. However, EE has been shown to increase neurogenesis in the dentate gyrus of 25-month-old male Wistar rats (32). Similarly, in 20–22-month-old rats, although neurogenesis decreased, access to EE enhanced new cell survival and increased behavioral spatial flexibility (70). These findings are relevant as neurogenic responses appear attenuated but not abolished in older animals, which is important for translational claims.

Regarding sex-specific effects, most of the reviewed studies include male mice and rats. Published reports indicate that between 61 and 72.6% of studies on EE are male-biased, limiting generalizability (79, 92). Nonetheless, gender-specific monoaminergic differences have been found when providing EE to female and male mice (54). In these animals, while males registered a significant reduction of hypothalamic 5-HIIA, no effect was observed in females. The authors concluded that the response in males was potentially associated with a down-regulation of the serotonergic function, while females might rely more on their sociability and rapid adaptation to the environment. However, sex-specific brain circuits are still under research. Further studies require the inclusion of both male and female animals in a single protocol to compare and understand the potential gender-related neurochemical response to EE.

Aggression has also been related to sex and strain, particularly when rodents are provided with EE, which is one of the potential negative outcomes that need to be considered when implementing an enrichment protocol for any animal species. A recent systematic review concluded that only 20% of studies associated EE with increased aggression in mice, particularly in males (93). However, it has been reported that EE can increase agonistic interactions in dominant male mice (94) and increase corticosterone levels in subdominant passive male mice as a result of distress (95). To ameliorate these effects, an alternative could be the selection of low-aggression strains (C57BL/6 and BALB/c mice or Sprague–Dawley rats) (93, 96) or selecting enrichment items that decrease aggression. It has been found that providing hiding devices and manipulable elements to rodents decreases agonistic interactions by eliciting interactive responses (86, 93), in contrast to objects that are easy to monopolize, such as running wheels (93).

### 5.5 Final considerations

Housing laboratory rodents in positively stimulating and complex environments has been shown to elicit beneficial neurochemical and neuroanatomical modifications. While reviewed studies on healthy rodents (non-animal models for diseases or human conditions) highlight the benefits and importance of EE, the limitations need to be considered to improve transparency and implementation of enrichment programs in laboratories. The wide heterogeneity of EE protocols limits direct comparisons between findings. It also complicates the introduction of EE protocols when studies do not systematically describe the number of items, rotation frequency, or duration. There is also a lack of direct social behavior quantification in many studies, and an absence of long-term follow-up after EE withdrawal. The variability in standard housing conditions is another critical aspect that is directly related to animal welfare and needs to be addressed, regardless of whether animals receive enrichment. Standardization of EE is challenging and is still a topic that requires research as it is a strategy that needs to be designed, implemented, and assessed considering physiological, behavioral, neurochemical, and neuroanatomical bases.

## 6 Conclusion

Enriched environments for rodents provide additional physical, sensorial, social, and cognitive stimulation. Constant stimulation has been shown to directly influence cognitive functions, including memory and learning. Cognitive functions elicited by EE, such as alertness, motivation, arousal, motor control, emotional regulation, and neuronal plasticity, are particularly dependent on the participation of the monoaminergic system. Monoamines, including DA, 5-HT, and NE, participate in the dynamic process to cope with environmental challenges. The response of the monoamines differs according to the neurotransmitter and the brain region. For example, DA concentrations have been shown to significantly decrease in the nucleus accumbens and the striatum of enriched animals. In contrast, significant increases have been reported in the hippocampus. Transporters and DA receptors also change their expression in response to EE, significantly decreasing in the mPFC. Regarding 5-HT, hippocampal concentrations increase in enriched rodents, while 5-HT metabolism decreases. A similar response is observed for NE in rats and mice exposed to EE, in whom significant increases have been reported in the hippocampus, striatum, and PTO. These neurochemical changes are also associated with modifications in the brain structure (e.g., increased cortical depth), neurogenesis in the dentate gyrus of the hippocampus, and increased synaptic excitability. These findings suggest that enriched environments changes monoamine regulation and influence neuroplasticity, improving the ability of laboratory rodents to habituate to novel environments and cope with several stimuli.

## References

[1] Renner MJ Rosenzweig MR. In: Renner MJ Rosenzweig MR, editors. The Neurobiology of Differential Experience. New York: Springer (1987)

[2] Diamond MC Krech D Rosenzweig MR. The effects of an enriched environment on the histology of the rat cerebral cortex. J Comp Neurol. (1964) 123:111–9. doi: 10.1002/cne.901230110 14199261

[3] Ratuski AS Weary DM. Environmental enrichment for rats and mice housed in laboratories: a Metareview. Animals. (2022) 12:414. doi: 10.3390/ani12040414, 35203123 PMC8868396

[4] Mieske P Hobbiesiefken U Fischer-Tenhagen C Heinl C Hohlbaum K Kahnau P . Bored at home?—a systematic review on the effect of environmental enrichment on the welfare of laboratory rats and mice. Front Vet Sci. (2022) 9:899219. doi: 10.3389/fvets.2022.899219, 36061113 PMC9435384

[5] Eckert MJ Abraham WC. "Effects of environmental enrichment exposure on synaptic transmission and plasticity in the Hippocampus". In: Belzung C Wigmore P, editors. Neurogenesis and Neural Plasticity. Current Topics in Behavioral Neurosciences. Berlin: Springer Berlin Heidelberg (2012). 10.1007/7854_2012_215 22798066

[6] Ohline SM Abraham WC. Environmental enrichment effects on synaptic and cellular physiology of hippocampal neurons. Neuropharmacology. (2019) 145:3–12. doi: 10.1016/j.neuropharm.2018.04.007 29634984

[7] van Praag H Kempermann G Gage FH. Neural consequences of enviromental enrichment. Nat Rev Neurosci. (2000) 1:191–8. doi: 10.1038/35044558 11257907

[8] Mochizuki-Kawai H Nakazawa S Oike H Kimoto H Tomita S Toyoshima M . Environmental restraint: a hidden risk factor for stress-induced depression in rats. IBRO Neurosci Reports. (2025) 18:754–8. doi: 10.1016/j.ibneur.2025.05.002, 40487593 PMC12144497

[9] Del Arco A Segovia G Canales JJ Garrido P de Blas M García-Verdugo JM . Environmental enrichment reduces the function of D1 dopamine receptors in the prefrontal cortex of the rat. J Neural Transm. (2007) 114:43–8. doi: 10.1007/s00702-006-0565-8 16955373

[10] National Research Council N. Guide for the care and use of Laboratory Animals. Washington: The National Academies Press (2011).

[11] Bayne K. Environmental enrichment and mouse models: current perspectives. Anim Model Exp Med. (2018) 1:82–90. doi: 10.1002/ame2.12015 PMC6388067 30891552

[12] Cait J Winder CB Mason GJ. How much “enrichment” is enough for laboratory rodents? A systematic review and meta-analysis re-assessing the impact of well-resourced cages on morbidity and mortality. Appl Anim Behav Sci. (2024) 278:106361. doi: 10.1016/j.applanim.2024.106361

[13] Clemenson GD Deng W Gage FH. Environmental enrichment and neurogenesis: from mice to humans. Curr Opin Behav Sci. (2015) 4:56–62. doi: 10.1016/j.cobeha.2015.02.005

[14] Domínguez-Oliva A Hernández-Avalos I Bueno-Nava A Chávez C Verduzco-Mendoza A Olmos-Hernández A . Environmental enrichment for laboratory rats and mice: endocrine, physiological, and behavioral benefits of meeting rodents’ biological needs. Front Vet Sci. (2025) 12:1622417. doi: 10.3389/FVETS.2025.1622417, 40708998 PMC12287651

[15] Smail MA Smith BL Nawreen N Herman JP. Differential impact of stress and environmental enrichment on corticolimbic circuits. Pharmacol Biochem Behav. (2020) 197:172993. doi: 10.1016/j.pbb.2020.172993, 32659243 PMC7484282

[16] Azizi SA. Monoamines: dopamine, norepinephrine, and serotonin, beyond modulation, “switches” that Alter the state of target networks. Neurosci. (2022) 28:121–43. doi: 10.1177/1073858420974336, 33292070

[17] Vallone D Picetti R Borrelli E. Structure and function of dopamine receptors. Neurosci Biobehav Rev. (2000) 24:125–32. doi: 10.1016/S0149-7634(99)00063-9, 10654668

[18] Stanwood GD. "Dopamine and stress". In: Fink G, editor. Stress: Physiology, Biochemistry, and Pathology. London: Academic Press (2019).

[19] Klein MO Battagello DS Cardoso AR Hauser DN Bittencourt JC Correa RG. Dopamine: functions, signaling, and association with neurological diseases. Cell Mol Neurobiol. (2019) 39:31–59. doi: 10.1007/s10571-018-0632-3, 30446950 PMC11469830

[20] Yildirim E Erol K Ulupinar E. Effects of sertraline on behavioral alterations caused by environmental enrichment and social isolation. Pharmacol Biochem Behav. (2012) 101:278–87. doi: 10.1016/j.pbb.2011.12.017, 22248860

[21] Brenes JC Rodríguez O Fornaguera J. Differential effect of environment enrichment and social isolation on depressive-like behavior, spontaneous activity and serotonin and norepinephrine concentration in prefrontal cortex and ventral striatum. Pharmacol Biochem Behav. (2008) 89:85–93. doi: 10.1016/j.pbb.2007.11.004, 18096212

[22] da Silva RPB Pinheiro IL da Silva RKB Moretti EC de Oliveira Neto OB Ferraz-Pereira K . Social isolation and post-weaning environmental enrichment effects on rat emotional behavior and serotonergic system. Int J Dev Neurosci. (2024) 84:265–80. doi: 10.1002/jdn.10324, 38526313

[23] Guan SZ Ji WJ Jiang Y Ning L Lian YL Liu JW. Enriched environment treatment remediated hippocampal monoamine neurotransmitters and emotional deficits in offspring induced by maternal chronic stress rat during pregnancy. Int J Clin Exp Med. (2017) 10:9963–75.

[24] Segovia G Del Arco A De Blas M Garrido P Mora F. Environmental enrichment increases the in vivo extracellular concentration of dopamine in the nucleus accumbens: a microdialysis study. J Neural Transm. (2010) 117:1123–30. doi: 10.1007/s00702-010-0447-y, 20706747

[25] Segovia G Del Arco A De Blas M Garrido P Mora F. Effects of an enriched environment on the release of dopamine in the prefrontal cortex produced by stress and on working memory during aging in the awake rat. Behav Brain Res. (2008) 187:304–11. doi: 10.1016/j.bbr.2007.09.024, 17977609

[26] Zhu J Green T Bardo MT Dwoskin LP. Environmental enrichment enhances sensitization to GBR 12935-induced activity and decreases dopamine transporter function in the medial prefrontal cortex. Behav Brain Res. (2004) 148:107–17. doi: 10.1016/S0166-4328(03)00190-6, 14684252

[27] Del Arco A Segovia G Garrido P Dde Blas M Mora F. Stress, prefrontal cortex and environmental enrichment: studies on dopamine and acetylcholine release and working memory performance in rats. Behav Brain Res. (2007) 176:267–73. doi: 10.1016/j.bbr.2006.10.006, 17097747

[28] Garrido P De Blas M Ronzoni G Cordero I Antón M Giné E . Differential effects of environmental enrichment and isolation housing on the hormonal and neurochemical responses to stress in the prefrontal cortex of the adult rat: relationship to working and emotional memories. J Neural Transm. (2013) 120:829–43. doi: 10.1007/s00702-012-0935-3, 23254925

[29] Ueda S Sakakibara S Yoshimoto K. Effect of long-lasting serotonin depletion on environmental enrichment-induced neurogenesis in adult rat hippocampus and spatial learning. Neuroscience. (2005) 135:395–402. doi: 10.1016/j.neuroscience.2005.06.065, 16125851

[30] Khalil MH. Environmental enrichment: a systematic review on the effect of a changing spatial complexity on hippocampal neurogenesis and plasticity in rodents, with considerations for translation to urban and built environments for humans. Front Neurosci. (2024) 18:1368411. doi: 10.3389/fnins.2024.1368411, 38919908 PMC11196820

[31] Brenes J Padilla M Fornaguera J. A detailed analysis of open-field habituation and behavioral and neurochemical antidepressant-like effects in postweaning enriched rats. Behav Brain Res. (2009) 197:125–37. doi: 10.1016/j.bbr.2008.08.014, 18786573

[32] Segovia G Yagüe AG García-Verdugo JM Mora F. Environmental enrichment promotes neurogenesis and changes the extracellular concentrations of glutamate and GABA in the hippocampus of aged rats. Brain Res Bull. (2006) 70:8–14. doi: 10.1016/j.brainresbull.2005.11.005, 16750477

[33] Faherty CJ Kerley D Smeyne RJ. A Golgi-cox morphological analysis of neuronal changes induced by environmental enrichment. Dev Brain Res. (2003) 141:55–61. doi: 10.1016/S0165-3806(02)00642-9 12644248

[34] Schrott L. Effect of training and environment on brain morphology and behavior. Acta Paediatr. (1997) 86:45–7. doi: 10.1111/j.1651-2227.1997.tb18344.x 9298792

[35] Nilsson M Perfilieva E Johansson U Orwar O Eriksson PS. Enriched environment increases neurogenesis in the adult rat dentate gyrus and improves spatial memory. J Neurobiol. (1999) 39:569–78. doi: 10.1002/(SICI)1097-4695(19990615)39:4<569::AID-NEU10>3.0.CO;2-F, 10380078

[36] Brown J Cooper-Kuhn CM Kempermann G Van Praag H Winkler J Gage FH . Enriched environment and physical activity stimulate hippocampal but not olfactory bulb neurogenesis. Eur J Neurosci. (2003) 17:2042–6. doi: 10.1046/j.1460-9568.2003.02647.x 12786970

[37] Browne CJ Fletcher PJ Zeeb FD. Responding for a conditioned reinforcer or unconditioned sensory reinforcer in mice: interactions with environmental enrichment, social isolation, and monoamine reuptake inhibitors. Psychopharmacology. (2016) 233:983–93. doi: 10.1007/s00213-015-4178-5 26690588

[38] Xu H Yang F. The interplay of dopamine metabolism abnormalities and mitochondrial defects in the pathogenesis of schizophrenia. Transl Psychiatry. (2022) 12:464. doi: 10.1038/s41398-022-02233-0, 36344514 PMC9640700

[39] Speranza L di Porzio U Viggiano D de Donato A Volpicelli F. Dopamine: the neuromodulator of long-term synaptic plasticity. Reward and Movement Control Cells. (2021) 10:735. doi: 10.3390/cells10040735, 33810328 PMC8066851

[40] Bowling SL Rowlett JK Bardo MT. The effect of environmental enrichment on amphetamine-stimulated locomotor activity, dopamine synthesis and dopamine release. Neuropharmacology. (1993) 32:885–93. doi: 10.1016/0028-3908(93)90144-R 8232791

[41] Kim M-S Yu JH Kim CH Choi JY Seo JH Lee M-Y . Environmental enrichment enhances synaptic plasticity by internalization of striatal dopamine transporters. J Cereb Blood Flow Metab. (2016) 36:2122–33. doi: 10.1177/0271678X15613525, 26661218 PMC5363660

[42] Winterfeld KT Teuchert-Noodt G Dawirs RR. Social environment alters both ontogeny of dopamine innervation of the medial prefrontal cortex and maturation of working memory in gerbils (*Meriones unguiculatus*). J Neurosci Res. (1998) 52:201–9. doi: 10.1002/(SICI)1097-4547(19980415)52:2<201::AID-JNR8>3.0.CO;2-E, 9579410

[43] Faherty CJ Raviie Shepherd K Herasimtschuk A Smeyne RJ. Environmental enrichment in adulthood eliminates neuronal death in experimental parkinsonism. Mol Brain Res. (2005) 134:170–9. doi: 10.1016/j.molbrainres.2004.08.008, 15790541

[44] Zhu J Apparsundaram S Bardo MT Dwoskin LP. Environmental enrichment decreases cell surface expression of the dopamine transporter in rat medial prefrontal cortex. J Neurochem. (2005) 93:1434–43. doi: 10.1111/j.1471-4159.2005.03130.x, 15935059

[45] Zhao S Piatkevich KD. Techniques for in vivo serotonin detection in the brain: state of the art. J Neurochem. (2023) 166:453–80. doi: 10.1111/jnc.15865, 37293767

[46] Pourhamzeh M Moravej FG Arabi M Shahriari E Mehrabi S Ward R . The roles of serotonin in neuropsychiatric disorders. Cell Mol Neurobiol. (2022) 42:1671–92. doi: 10.1007/s10571-021-01064-9, 33651238 PMC11421740

[47] Rasmuson S Olsson T Henriksson BG Kelly PA Holmes MC Seckl JR . Environmental enrichment selectively increases 5-HT1A receptor mRNA expression and binding in the rat hippocampus. Mol Brain Res. (1998) 53:285–90. doi: 10.1016/S0169-328X(97)00317-3 9473697

[48] Galani R Berthel M-C Lazarus C Majchrzak M Barbelivien A Kelche C . The behavioral effects of enriched housing are not altered by serotonin depletion but enrichment alters hippocampal neurochemistry. Neurobiol Learn Mem. (2007) 88:1–10. doi: 10.1016/j.nlm.2007.03.009 17493843

[49] Naka F Shiga T Yaguchi M Okado N. An enriched environment increases noradrenaline concentration in the mouse brain. Brain Res. (2002) 924:124–6. doi: 10.1016/S0006-8993(01)03257-7, 11744005

[50] Breton-Provencher V Drummond GT Sur M. Locus Coeruleus norepinephrine in learned behavior: anatomical modularity and spatiotemporal integration in targets. Front. Neural Circuits. (2021) 15:7. doi: 10.3389/fncir.2021.638007, 34163331 PMC8215268

[51] Hussain LS Reddy V Maani CV. Physiology, Noradrenergic System. Florida: StatPearls Publishing (2023). 31082021

[52] Storey J Robertson D Beattie J Reid I Mitchell S Balfour D. Behavioural and neurochemical responses evoked by repeated exposure to an elevated open platform. Behav Brain Res. (2006) 166:220–9. doi: 10.1016/j.bbr.2005.08.002, 16150498

[53] Slattery DA Cryan JF. Using the rat forced swim test to assess antidepressant-like activity in rodents. Nat Protoc. (2012) 7:1009–14. doi: 10.1038/nprot.2012.044 22555240

[54] Chourbaji S Hörtnagl H Molteni R Riva MA Gass P Hellweg R. The impact of environmental enrichment on sex-specific neurochemical circuitries – effects on brain-derived neurotrophic factor and the serotonergic system. Neuroscience. (2012) 220:267–76. doi: 10.1016/j.neuroscience.2012.06.016, 22710068

[55] Glikmann-Johnston Y Saling MM Reutens DC Stout JC. Hippocampal 5-HT1A receptor and spatial learning and memory. Front Pharmacol. (2015) 6:289. doi: 10.3389/fphar.2015.00289, 26696889 PMC4674558

[56] Detke MJ Rickels M Lucki I. Active behaviors in the rat forced swimming test differentially produced by serotonergic and noradrenergic antidepressants. Psychopharmacology. (1995) 121:66–72. doi: 10.1007/BF02245592, 8539342

[57] Brenes JC Fornaguera J Sequeira-Cordero A. Environmental enrichment and physical exercise attenuate the depressive-like effects induced by social isolation stress in rats. Front Pharmacol. (2020) 11:804. doi: 10.3389/fphar.2020.00804, 32547399 PMC7272682

[58] Harris AP D’Eath RB Healy SD. Environmental enrichment enhances spatial cognition in rats by reducing thigmotaxis (wall hugging) during testing. Anim Behav. (2009) 77:1459–64. doi: 10.1016/j.anbehav.2009.02.019

[59] Yuki N Sawamura T Mori A Yamaguchi H Horii Y Shiina T . Involvement of the hypothalamus–raphe magnus–spinal defecation center axis in stress-induced defecation in rats. Commun Biol. (2026) 9:411. doi: 10.1038/s42003-026-09779-5, 41922653 PMC13043784

[60] Kalueff AV Stewart AM Song C Berridge KC Graybiel AM Fentress JC. Neurobiology of rodent self-grooming and its value for translational neuroscience. Nat Rev Neurosci. (2016) 17:45–59. doi: 10.1038/nrn.2015.8, 26675822 PMC4840777

[61] Givon L Edut S Klavir O. The role of fear and dopamine-striatal pathways in grooming. Neuropharmacology. (2025) 269:110323. doi: 10.1016/j.neuropharm.2025.110323, 39880328

[62] Markham JA Greenough WT. Experience-driven brain plasticity: beyond the synapse. Neuron Glia Biol. (2004) 1:351–63. doi: 10.1017/S1740925X05000219, 16921405 PMC1550735

[63] Rosenzweig MR Krech D Bennett EL Diamond MC. Effects of environmental complexity and training on brain chemistry and anatomy: a replication and extension. J Comp Physiol Psychol. (1962) 55:429–37. doi: 10.1037/h0041137, 14494091

[64] Comery TA Stamoudis CX Irwin SA Greenough WT. Increased density of multiple-head dendritic spines on medium-sized spiny neurons of the striatum in rats reared in a complex environment. Neurobiol Learn Mem. (1996) 66:93–6. doi: 10.1006/nlme.1996.0049, 8946401

[65] Greenough WT Volkmar FR Juraska JM. Effects of rearing complexity on dendritic branching in frontolateral and temporal cortex of the rat. Exp Neurol. (1973) 41:371–8. doi: 10.1016/0014-4886(73)90278-1, 4126876

[66] Diamond MC Rosenzweig MR Bennett EL Lindner B Lyon L. Effects of environmental enrichment and impoverishment on rat cerebral cortex. J Neurobiol. (1972) 3:47–64. doi: 10.1002/neu.480030105, 5028293

[67] Diamond MC Law F Rhodes H Lindner B Rosenzweig MR Krech D . Increases in cortical depth and glia numbers in rats subjected to enriched environment. J Comp Neurol. (1966) 128:117–25. doi: 10.1002/cne.901280110, 4165855

[68] Scholz J Allemang-Grand R Dazai J Lerch JP. Environmental enrichment is associated with rapid volumetric brain changes in adult mice. NeuroImage. (2015) 109:190–8. doi: 10.1016/j.neuroimage.2015.01.027, 25595504

[69] Barros W David M Souza A Silva M Matos R. Can the effects of environmental enrichment modulate BDNF expression in hippocampal plasticity? A systematic review of animal studies. Synapse. (2019) 73:e22103. doi: 10.1002/syn.22103 31056812

[70] Speisman RB Kumar A Rani A Pastoriza JM Severance JE Foster TC . Environmental enrichment restores neurogenesis and rapid acquisition in aged rats. Neurobiol Aging. (2013) 34:263–74. doi: 10.1016/j.neurobiolaging.2012.05.023, 22795793 PMC3480541

[71] Monteiro BMM Moreira FA Massensini AR Moraes MFD Pereira GS. Enriched environment increases neurogenesis and improves social memory persistence in socially isolated adult mice. Hippocampus. (2014) 24:239–48. doi: 10.1002/hipo.22218, 24123782

[72] Fabel K. Additive effects of physical exercise and environmental enrichment on adult hippocampal neurogenesis in mice. Front Neurosci. (2009) 3:50. doi: 10.3389/neuro.22.002.2009, 20582277 PMC2858601

[73] van Praag H Shubert T Zhao C Gage FH. Exercise enhances learning and hippocampal neurogenesis in aged mice. J Neurosci. (2005) 25:8680–5. doi: 10.1523/JNEUROSCI.1731-05.2005, 16177036 PMC1360197

[74] Kempermann G Kuhn HG Gage FH. More hippocampal neurons in adult mice living in an enriched environment. Nature. (1997) 386:493–5. doi: 10.1038/386493a0, 9087407

[75] Stein LR O’Dell KA Funatsu M Zorumski CF Izumi Y. Short-term environmental enrichment enhances synaptic plasticity in hippocampal slices from aged rats. Neuroscience. (2016) 329:294–305. doi: 10.1016/j.neuroscience.2016.05.020, 27208617 PMC4924801

[76] Green EJ Greenough WT. Altered synaptic transmission in dentate gyrus of rats reared in complex environments: evidence from hippocampal slices maintained in vitro. J Neurophysiol. (1986) 55:739–50. doi: 10.1152/jn.1986.55.4.739, 3009728

[77] Irvine GI Logan B Eckert M Abraham WC. Enriched environment exposure regulates excitability, synaptic transmission, and LTP in the dentate gyrus of freely moving rats. Hippocampus. (2006) 16:149–60. doi: 10.1002/hipo.20142, 16261558

[78] European Union E. Directive 2010/63/EU of the European parliament and of the council on the protection of animals used for scientific purposes. (2010) Available online at: https://eur-lex.europa.eu/eli/dir/2010/63/oj/eng [Accessed April 5, 2026]

[79] Simpson J Kelly JP. The impact of environmental enrichment in laboratory rats—behavioral and neurochemical aspects. Behav Brain Res. (2011) 222:246–64. doi: 10.1016/j.bbr.2011.04.002, 21504762

[80] Toth LA Kregel K Leon L Musch TI. Environmental enrichment of laboratory rodents: the answer depends on the question. Comp Med. (2011) 61:314–21. 22330246 PMC3155397

[81] Van Loo PL Mol JA Koolhaas JM Van Zutphen BF Baumans V. Modulation of aggression in male mice: influence of group size and cage size. Physiol Behav. (2001) 72:675–83. doi: 10.1016/S0031-9384(01)00425-5, 11336999

[82] Jirkof P Bratcher N Medina L Strasburg D Ebert P Gaskill BN. The effect of group size, age and handling frequency on inter-male aggression in CD 1 mice. Sci Rep. (2020) 10:2253. doi: 10.1038/s41598-020-59012-4 32042065 PMC7010790

[83] Patterson-Kane EP Hunt M Harper D. Short communication: rat’s demand for group size. J Appl Anim Welf Sci. (2004) 7:267–72. doi: 10.1207/s15327604jaws0704_4, 15857812

[84] Rivalan M Alonso L Mosienko V Bey P Hyde A Bader M . Serotonin drives aggression and social behaviors of laboratory male mice in a semi-natural environment. Front Behav Neurosci. (2024) 18:1450540. doi: 10.3389/fnbeh.2024.1450540, 39359324 PMC11446219

[85] de Lima APN Sandini TM Reis-Silva TM Massoco CO. Long-lasting monoaminergic and behavioral dysfunctions in a mice model of socio-environmental stress during adolescence. Behav Brain Res. (2017) 317:132–40. doi: 10.1016/j.bbr.2016.09.024 27641324

[86] Hutchinson E Avery A VandeWoude S. Environmental enrichment for laboratory rodents. ILAR J. (2005) 46:148–61. doi: 10.1093/ilar.46.2.148 15775024

[87] Miranda M Navas MC Zanoni Saad MB Piromalli Girado D Weisstaub N Bekinschtein P. Environmental enrichment in middle age rats improves spatial and object memory discrimination deficits. Front Behav Neurosci. (2024) 18:1478656. doi: 10.3389/fnbeh.2024.1478656, 39494036 PMC11528545

[88] Bennett J McRae P Levy L Frick K. Long-term continuous, but not daily, environmental enrichment reduces spatial memory decline in aged male mice. Neurobiol Learn Mem. (2006) 85:139–52. doi: 10.1016/j.nlm.2005.09.003 16256380

[89] Rojas-Carvajal M Sequeira-Cordero A Brenes JC. Neurobehavioral effects of restricted and unpredictable environmental enrichment in rats. Front Pharmacol. (2020) 11:674. doi: 10.3389/fphar.2020.00674, 32477137 PMC7235364

[90] Cloutier S Baker C Wahl K Panksepp J Newberry RC. Playful handling as social enrichment for individually and group-housed laboratory rats. Appl Anim Behav Sci. (2013) 143:85–95. doi: 10.1016/j.applanim.2012.10.006

[91] Tang AC. Neonatal exposure to novel environment enhances hippocampal-dependent memory function during infancy and adulthood. Learn Mem. (2001) 8:257–64. doi: 10.1101/lm.43101, 11584072 PMC311382

[92] Cait J Cait A Scott RW Winder CB Mason GJ. Conventional laboratory housing increases morbidity and mortality in research rodents: results of a meta-analysis. BMC Biol. (2022) 20:15. doi: 10.1186/s12915-021-01184-0, 35022024 PMC8756709

[93] Weber EM Zidar J Ewaldsson B Askevik K Udén E Svensk E . Aggression in group-housed male mice: a systematic review. Animals. (2022) 13:143. doi: 10.3390/ani13010143, 36611751 PMC9817818

[94] Haemisch A Gärtner K. Effects of cage enrichment on territorial aggression and stress physiology in male laboratory mice. Acta Physiol Scand Suppl. (1997) 640:73–6. 9401611

[95] Haemisch A Voss T Gärtner K. Effects of environmental enrichment on aggressive behavior, dominance hierarchies, and endocrine states in male DBA/2J mice. Physiol Behav. (1994) 56:1041–8. doi: 10.1016/0031-9384(94)90341-7, 7824569

[96] Snyder B Duong P Tenkorang M Wilson EN Cunningham RL. Rat strain and housing conditions Alter oxidative stress and hormone responses to chronic intermittent hypoxia. Front Physiol. (2018) 9:1554. doi: 10.3389/fphys.2018.01554, 30459637 PMC6232418

