# Supplementary material for: Influence of environmental enrichment on rodents’ brains: neurochemical and neuroanatomical aspects
Source: Front Vet Sci. 2026 May 7;13:1767287. doi: 10.3389/fvets.2026.1767287 (PMC13189735; doi:10.3389/fvets.2026.1767287)
Supplement: Supplementary file 1 [file Table_1.docx]

Supplementary Material

Supplementary Table 1. Experimental characteristics and main changes in the monoaminergic system of rodents exposed to EE.

| Species | Age  (PND) | Sex (M/F) | Standard condition | Enriched condition | | | | | Rotation frequency | Light/dark phase | Duration | Outcome | Ref. |
| --- | --- | --- | --- | --- | --- | --- | --- | --- | --- | --- | --- | --- | --- |
|  |  |  |  | Social | Structural | Nutri | Sens | Cogn |  |  |  |  |  |
| Rat | 30 | M | Standard cage (26.5 × 42 × 18 cm)  3 rats/cage | Group  3 rats | Large cage  (120×70×100 cm)  Non-chewable objects  PVC tubes (2) | X | X | X | Every 2 d | Dark | 84 d | PFC:  ↑ 5-HT  Ventral striatum:  ↑ NE | Brenes et al. (21) |
| Rat | 28 | M | Standard cage (26.5 × 42 × 18 cm)  3 rats/cage | Group  3 rats | Large cage  (120×70×100 cm)  Non-chewable objects  PVC tubes (2) | X | X | X | Every 1–2 d | Dark | 42 d | Hipp:  ↑ 5-HT  ↓ 5-HT turnover  ↑ NE | Brenes et al. (31) |
| Rat | 21 | M | Standard cage  Housed singly  Pine bedding | Group  12–13 rats | Large cage  (94×94×51cm)  Toys | X | X | X | Daily | NS | NS | Nucleus accumbens and striatum:  ↓ DA | Bowling et al. (40) |
| Mice | 28 | M  F | Type III cage (42×25×15 cm)  Bedding material  6 rats/cage | Group  6 rats | Plastic tunnels  Plastic igloos  Nesting material (tissue) | X | X | X | Weekly | NS | 7–8 w | Hipp:  ↓ 5-HIIA | Chourbaji et al. (54) |
| Rat | ~90 | M | Plexiglas cage  (55×35×20 cm)  1 rat/cage | Group  10–12 rats | Large cage  (120×100×60 cm)  Running wheel (2)  Plastic tunnels  Elevated platform (1)  Toys | X | X | X | 5–6 d | Dark | 12 m | mPFC during handling stress:  – DA  ↓ ACh | Del Arco et al. (27) |
| Rat | ~90 | M | Plexiglas cage  (55×35×20 cm)  1 rat/cage | Group  10–12 rats | Large cage  (120×100×60 cm)  Running wheel (2)  Plastic tunnels  Elevated platform (1)  Toys | X | X | X | 5–6 d | Dark | 90 d | PFC:  ↓ DA1 receptor | Del Arco et al. (9) |
| Rat | 84 | F | Makrolon cage  (42×26×15 cm)  2 rats/cage | Group  10–12 rats | Large wire-mesh cage  (112×40×40 cm)  Various objects | X | X | X | Daily | NS | 30 d | Ventral Hipp:  ↓ 5-HT  ↑ 5-HT turnover  Dorsal Hipp:  ↑ NE | Galani et al. (48) |
| Rat | ~90 | M | Plexiglas cage  (55×35×20 cm)  1 rat/cage | Group  10–12 rats | Large cage  (120×100×60 cm)  Running wheel (2)  Tunnels  Elevated platform (1)  Objects | X | X | X | 5–6 d | Dark | 3 m | PFC during restraint stress:  ↑ DA, but EE ameliorated this effect | Garrido et al. (28) |
| Rat | 21 | M  F | Standard cage  (60×40×25 cm)  4 rats/cage | Group  8 rats | Large wire-mesh cage  (90×50×60 cm)  Ramps  Two levels  Running wheel (1)  Shelter (1)  Plastic color toys  Chain  Swing  Tunnels | X | X | X | Weekly | NS | 30 d | Hipp:  ↑ DA  ↑ 5-HT  ↑ NE | Guan et al. (23) |
| Mice | 42 | NS | Standard cage  (27×22.5×14 cm^3^)  Bedding | Group  10 mice | Large cage  (86×76×31 cm^3^)  Tunnels (4)  Shelters (2)  Toys (6)  Running wheels (4) | X | X | X | NS | NS | 2 m | mPFC:  ↓ DAT | Kim et al. (41) |
| Mice | 28 | M | Standard plexiglass cage (30×18×15 cm)  3–4 mice/cage | Group  11 mice | Large cage  (90×90×30 cm)  Plastic tubes  Small house (1)  Ladder (1)  Running wheel  Toys | X | X | X | Access 3 h daily  Changed every 2 d | NS | 40 d | PTO cortex:  ↑ NE | Naka et al. (49) |
| Rat | 52 | M | Plexiglass cage  (16.5×22.5×13.5 cm)  1 rat/cage | Group  4 rats | Large wire-mesh cage  (100×60×35 cm)  Objects  Toys | X | X | X | Twice a week | NS | 30 d | Dorsal Hipp:  ↑ 5HT_1A_ | Rasmuson et al. (47) |
| Rat | ~90 | M | Plexiglass cage (55×35×20 cm)  1 rat/cage | Group  10–12 rats | Large cage  (120×100×60 cm)  Running wheels (2)  Plastic tunnels  Elevated platform (1)  Toys | X | X | X | 5–6 d | Dark | 6 m | Nucleus accumbens:  ↑ DA | Segovia et al. (24) |
| Rat | ~90 | M | Plexiglass cage (55×35×20 cm)  1 rat/cage | Group  10–12 rats | Large cage  (120×100×60 cm)  Running wheels (2)  Plastic tunnels  Elevated platform (1)  Toys | X | X | X | 5–6 d | Dark | 6 m | PFC during handling stress:  ↑ DA, but EE ameliorated this effect | Segovia et al. (25) |
| Gerbil | 90 | M | Standard cage  1 rat/cage | Group | Semi-natural cage  Tunnels  Branches  Hiding places | X | X | X | NS | NS | 60 d | mPFC:  ↑ DA innervation | Winterfeld et al. (42) |
| Rat | 21 | M | Wire mesh hanging cage  (25×18×17 cm)  1 rat/cage | Group  8–12 rats | Large cage  (120×60×45 cm)  Non-chewable plastic objects (14) | X | X | X | One-half of the objects daily | Light | 1 m | mPFC, striatum, and nucleus accumbens:  – DA  mPFC:  ↓ DOPAC | Zhu et al. (26) |
| Rat | 21 | M | Wire mesh hanging cage  (25×18×17 cm)  1 rat/cage | Group  8–12 rats | Large cage  (120×60×45 cm)  Non-chewable objects (14) | X | X | X | One-half of the objects daily | NS | 1 m | mPFC:  ↓ DAT | Zhu et al. (44) |

Abbreviations: 5-HT: serotonin; 5-HIIA: 5-hydroxyindoleacetic acid; ACh: acetylcholine; Cogn: cognitive; d: days; DA: dopamine; DAT: dopamine transporter; DOPAC: 3,4-Dihydroxyphenylacetic acid; EE: environmental enrichment; F: female; Hipp: hippocampus; M: male; m: month; mPFC: medial prefrontal cortex; NE: norepinephrine; NS: not specified; Nutri: nutritional; PFC: prefrontal cortex; PND: postnatal days; Ref: references; Sens: sensorial; w: weeks.
