# Supplementary material for: Influence of environmental enrichment on rodents’ brains: neurochemical and neuroanatomical aspects
Source: Front Vet Sci. 2026 May 7;13:1767287. doi: 10.3389/fvets.2026.1767287 (PMC13189735; doi:10.3389/fvets.2026.1767287)
Supplement: Supplementary file 2 [file Table_2.docx]

Supplementary Material

Supplementary Table 2. Main changes in neuroanatomical and neurotransmission characteristics in rodents exposed to EE.

| Species | Age  (PND) | Sex  (M/F) | Standard condition | Enriched conditions | | | | | Rotation frequency | Light/dark phase | Duration | Outcome | Refer. |
| --- | --- | --- | --- | --- | --- | --- | --- | --- | --- | --- | --- | --- | --- |
|  |  |  |  | Social | Structural | Nutri | Sens | Cogn |  |  |  |  |  |
| Rats | 25 | M | Small cage  (34×20×20 cm)  1 rat/cage | Group  10–12 rats | Large cage  (70×70×46 cm)  Ladders  Tunnels  Swings  Other objects | X | X | Hebb-Williams maze | Access to the mazes for 30 min daily | NS | 30 d | ↑ Cortical depth | Diamond et al. (66) |
| Rat | 25 | M | Small cages  (11×8×8 in)  1 rat/cage | Group  10 rats | Large cage  (25×25×18 in)  Wooden toys (2) | X | X | Hebb-Williams maze  Training  Lashley III maze  Dashiell maze  Krech Hypothesis apparatus | Toys changed daily  Access to the mazes for 30 min daily | NS | 30 d | ↑ Cortical depth  ↑ Visual cortex depth | Diamond et al. (2,67) |
| Mice | 21 | M | Standard cage  (12 × 6 inches)  4 mice/cage | Group  14 mice | Large cage  (1×1 m)  Running wheels (2)  Nesting material  Tunnels | X | X | X | Weekly | NS | 17 w | Hipp pyramidal cells in CA1  ↑ Cell volume of  ↑ Dendrite length | Faherty et al. (33) |
| Rat | 22 | M | Translucent tubs  1 rat | Groups  10–15 rats | Large cage  (80×80×90 cm)  Objects  Separated area (1.2 m^2^) | X | X | X | Objects rotated daily  EE: 30–60 min in the separated area daily | Light | 25–34 d | ↑ Basal synaptic transmission in the Hipp | Green and Greenough (76) |
| Rat | 70–182 | M | Hanging metal cage  (18×24×17 cm)  1 rat/cage | X | Large cage  Plastic drainpipes  Tunnels  Ladders  Children’s toys | Novel food (Kellogg’s Coco Pops) | X | X | Objects rotated daily  EE: 1 h or overnight exposure | Light | 19 d | ↑ Excitatory postsynaptic potentials  ↑ Population spikes | Irvine et al. (77) |
| Mice | 56–84 | M | Standard plastic cage  (28×17×12 cm^3^)  Isolated and 5 mice/cage | Isolated and groups of 5 rats/cage | Large cage  (40×33×16 cm^3^)  Plastic shelter (1)  Cardboard rolls (2)  Toys (3) Ribbons | X | X | X | NS | Light | 7 d | ↑ Neurogenesis in the dentate gyrus | Monteiro et al. (71) |
| Rat | 63 | F | Standard cages  (45×22×18 cm)  1 rat/cage | Group  6 rats | Large cage  (100×60×60 cm)  Climbing ladders  Platforms  Paper  Nesting material  Cardboard nest | Popcorn  Apples | X | X | 3–4 d | NS | 8 w | ↑ BrdU-positive cells | Nilsson et al. (35) |
| Mice | 49 | M | Standard cage  5 mice/cage | Group  7 mice | Double Decker Rat IVC cage  (462×403×404 cm)  Dome (1)  Running wheel (1) | X | X | Three-level maze | Every 3 d | NS | 3 w | ↑ Somatosensory cortex  ↑ Visual cortex  ↑ Ventral Hipp  ↑ Hypothalamus  ↑ VTA  ↑ Striatum | Scholz et al. (68) |
| Wat | 2 m  25 m | M | Wire mesh cage  1 rat/cage | Group  8 rats | Large cage  (120×100×60 cm)  Running wheel (2)  Plastic tunnels  Elevated platform (1)  Toys | X | X | X | 3–4 d | Dark | 8 w | ↑ BrdU-positive cells in the dentate gyrus | Segovia et al. (32) |
| Rat | 140–154 | M | 1 rat/cage | Pair | Large wooden box  Empty water maze tank  Plastic tubes  Balls  Various objects | X | X | X | Access to EE for 2–3 h daily  Objects rotated daily | NS | 10 w | ↑ Neurogenesis in the dentate gyrus | Speisman et al. (70) |
| Rat | 21 m | M | Standard cage  1 rat/cage | X | Large cage  (15×30×30 in)  Toys  Various objects | X | X | X | Access to EE for 3 h daily  Objects rotated daily |  | 3 w | EE elicited long-term potentiation and long-term depression | Stein et al. (75) |
| Rat | 21 | M | Standard cage  (45×30×20 cm)  2 rats/cage | Group  2 rats | Large cage  (80×80×40 cm)  Running wheel  Toys  Platform  Boxes  Nesting material | X | X | X | Daily | NS | 4 w | ↑ BrdU-positive cells in the Hipp | Ueda et al. (29) |

Abbreviations: BrdU: 5-bromo-2'-deoxyuridine; Cogn: cognitive; d: days; F: female; Hipp: hippocampus; M: male; Nutri: nutritional; NS: not specified; PND: postnatal days; Ref: references; Sens: sensorial; VTA: ventral tegmental area; w: weeks.
